# Characterization of the Zika virus induced small RNA response in *Aedes aegypti* cells

**DOI:** 10.1371/journal.pntd.0006010

**Published:** 2017-10-17

**Authors:** Margus Varjak, Claire L. Donald, Timothy J. Mottram, Vattipally B. Sreenu, Andres Merits, Kevin Maringer, Esther Schnettler, Alain Kohl

**Affiliations:** 1 MRC-University of Glasgow Centre for Virus Research, Glasgow, Scotland, United Kingdom; 2 Institute of Technology, University of Tartu, Nooruse 1, Tartu, Estonia; 3 Department of Microbial Sciences, Faculty of Health and Medical Sciences, University of Surrey, Guildford, United Kingdom; 4 Bernhard-Nocht-Institute for Tropical Medicine, Bernhard-Nocht-Strasse, Hamburg, Germany; Colorado State University, UNITED STATES

## Abstract

RNA interference (RNAi) controls arbovirus infections in mosquitoes. Two different RNAi pathways are involved in antiviral responses: the PIWI-interacting RNA (piRNA) and exogenous short interfering RNA (exo-siRNA) pathways, which are characterized by the production of virus-derived small RNAs of 25–29 and 21 nucleotides, respectively. The exo-siRNA pathway is considered to be the key mosquito antiviral response mechanism. In *Aedes aegypti*-derived cells, Zika virus (ZIKV)-specific siRNAs were produced and loaded into the exo-siRNA pathway effector protein Argonaute 2 (Ago2); although the knockdown of Ago2 did not enhance virus replication. Enhanced ZIKV replication was observed in a Dcr2-knockout cell line suggesting that the exo-siRNA pathway is implicated in the antiviral response. Although ZIKV-specific piRNA-sized small RNAs were detected, these lacked the characteristic piRNA ping-pong signature motif and were bound to Ago3 but not Piwi5 or Piwi6. Silencing of PIWI proteins indicated that the knockdown of Ago3, Piwi5 or Piwi6 did not enhance ZIKV replication and only Piwi4 displayed antiviral activity. We also report that the expression of ZIKV capsid (C) protein amplified the replication of a reporter alphavirus; although, unlike yellow fever virus C protein, it does not inhibit the exo-siRNA pathway. Our findings elucidate ZIKV-mosquito RNAi interactions that are important for understanding its spread.

## Author summary

The recent outbreak of Zika virus (ZIKV) in the Americas has resulted in a severe threat to public health. ZIKV is transmitted by *Aedes aegypti* mosquitoes, thus it is important to understand virus-vector interactions. Analysis of ZIKV infection in mosquito cells indicated that two RNA interference pathways are involved during infection: the exogenous short-interfering (si)RNA (exo-siRNA) and PIWI-interacting (pi)RNA pathways. If Dcr2, an enzyme responsible for cleaving dsRNA into siRNAs, is knocked out, ZIKV replication is increased compared to control cells. However, the knockdown of Ago2 expression had no significant enhancing effect on ZIKV replication. In the case of the PIWI pathway, only the Piwi4 protein was found to have significant antiviral activity. Furthermore, unlike the capsid (C) protein of yellow fever virus, ZIKV capsid protein does not suppress the siRNA pathway. These results suggest that ZIKV has mechanisms to evade mosquito innate immunity and it is therefore important to understand these virus-vector interactions and the implications they have on transmission.

## Introduction

Zika virus (ZIKV) is an arbovirus belonging to the family *Flaviviridae*, genus *Flavivirus*. The ZIKV genome is a single-stranded, positive-sense RNA molecule that demonstrates typical flavivirus organization—with a single open reading frame encoding the structural and non-structural proteins, flanked by 5’ and 3’ non coding regions [1,2]. The emergence of the virus in the Americas at the beginning of 2015, in addition to its links to Guillain-Barré syndrome as well as microcephaly, placed the virus firmly in the spotlight [3–5].

Arboviruses infect arthropod vectors and induce antiviral responses that control their replication. The most prominent of these are the RNA interference (RNAi) pathways [6,7]. In *Aedes aegypti* mosquitoes, known to be the key vector for ZIKV transmission [8], there are two RNAi pathways associated with antiviral responses: the exogenous small interfering (si)RNA (exo-siRNA) and the PIWI-interacting (pi)RNA (piRNA) pathway. Much of our understanding of mosquito antiviral RNAi is based on comparisons with the *Drosophila melanogaster* model. Virus RNA replication results in the synthesis of double-stranded RNA (dsRNA) replication intermediates that are cleaved into 21 nucleotide (nt) long virus-specific siRNAs (vsiRNAs) by Dicer 2 (Dcr2). In turn, vsiRNAs are loaded into the Argonaute 2 (Ago2) protein, which is part of the RNA-induced silencing complex (RISC). It is presumed that one strand of the vsiRNA duplex is degraded and the remaining strand guides Ago2 to complementary viral RNA, resulting in the cleavage and degradation of the target [9–20]. The production of vsiRNAs has been identified in arbovirus infected mosquitoes as well as in their derived cell lines [21–30].

Virus-specific piRNAs (vpiRNA) have also been described in arbovirus infected mosquitoes and derived cell lines [20–22,24,28,31,32]. These are 25–29 nt in length and are produced through a so-called ‘‘ping-pong” amplification loop which gives the vpiRNAs specific molecular signatures: primary-type piRNAs have a uridine at position 1 [U1] and secondary piRNAs have an adenine at position 10 [A10]. The *Ae*. *aegypti* genome encodes seven PIWI proteins (Piwi1-7) and a single Ago3 protein involved in this pathway [24,33,34]. The role of piRNAs in the control of arbovirus infection is enigmatic and although piRNAs have been suggested to be antiviral, this has not been directly demonstrated. The only PIWI protein with antiviral activity in *Ae*. *aegypti* is Piwi4. However, Piwi4 does not bind piRNAs, nor is it involved in the production of virus-specific piRNAs [24,34,35]. Previous studies have shown that Ago2 silencing in mosquito-derived cells or mosquitoes increased replication of arboviruses of the *Togaviridae* (genus *Alphavirus*), *Flaviviridae* and *Bunyaviridae* (genus *Orthobunyavirus*) families [23,24,31,35–37] following pioneering work on this RNAi effector protein in *D*. *melanogaster* [38]. Silencing of Piwi4 has only been shown to result in the upregulation of the replication of the alphavirus Semliki Forest virus (SFV) and the bunyaviruses Bunyamwera virus (BUNV) and Rift Valley fever virus (RVFV) [24,31,39].

Here we studied ZIKV interactions with the RNAi response of *Ae*. *aegypti* mosquito cells. We found that ZIKV induced the production of both virus-specific siRNAs and piRNAs, although vpiRNAs lack the specific signature expected of such small RNAs. Furthermore, our findings indicate that although vsiRNAs were loaded into Ago2, silencing of this protein did not enhance virus replication. Indeed, increased replication in Dcr2 knockout cell lines was the main indicator for an antiviral role of the exo-siRNA pathway. With the exception of Piwi4, the knockdown of PIWI proteins also did not enhance virus replication. Moreover, ZIKV capsid protein (C) amplified the replication of an alphavirus-based reporter system but did not display exo-siRNA pathway inhibition, unlike that of another flavivirus, yellow fever virus (YFV) [40]. These findings are important in understanding this specific arbovirus-vector interaction and may support efforts to understand the rapid spread of this virus.

## Materials and methods

### Plasmids

V5-tagged proteins were expressed under the control of the *Ae*. *aegypti* polyubiquitin promoter in stably transfected cell lines and the plasmids pIZ-Fluc and pAc1-Rluc have been described previously [41]. Plasmid, pCMV-SFV6(3H)-*RLuc*-2SG, containing reporter virus cDNA based on SFV strain 6 [42] was used for cloning purposes. The reporter virus expresses *Renilla* luciferase from a duplicated nsP2 cleavage site located between nsP3 and nsP4 proteins. It also contains a duplicated subgenomic promoter allowing it to also express either the tombusvirus RNAi inhibitor p19, ZIKV capsid protein C or eGFP. The infectious clone-containing plasmids are referred to as pCMV-SFV6(3H)-*RLuc*-2SG-p19, pCMV-SFV6(3H)-*RLuc*-2SG-ZIKV_C or pCMV-SFV6(3H)-*RLuc*-2SG-eGFP, respectively. Firefly luciferase-expressing reporter virus SFV(3H)-*FFLuc* has been previously used and is based of SFV strain 4 [41]. Rescue of SFV from cDNA and titration have been previously described [41,43].

### Cells and ZIKV

*Ae*. *aegypti*-derived Aag2 cells [31] (received from P. Eggleston, Keele University, UK), were grown in L-15+Glutamax (Life Technologies) supplemented with 10% Tryptose Phosphate Broth (TPB, Life Technologies), 10% fetal bovine serum (FBS, Life Technologies), and penicillin-streptomycin (final concentration 100 units/mL, 100 μg/mL respectively, Life Technologies). *Ae*. *aegypti*-derived Dcr2 KO cells AF319 and their parental cell line, AF5 (produced previously by the authors, see [41]) were grown in same conditions as Aag2 cells. The media for growing Aag2-based cell lines expressing V5-tagged proteins (established previously by the authors, see [41]) also included zeocin (final concentration of 100 μg/ml). A549/BVDV-Npro cells (stably expressing Npro protein of bovine viral diarrhea virus, which induces degradation of IRF3 and thus optimized for virus growth; provided by R. E. Randall, University of St Andrews, UK) [44] were grown in DMEM (Life Technologies) supplemented with 10% FBS, puromycin (2 μg/ml) and penicillin-streptomycin (final concentration 100 units/mL, 100 μg/mL, respectively) at 37°C/ 5% CO_2_; these cells were used for plaque titration of ZIKV. The Brazilian ZIKV strain PE243 used in the study has been described previously by the authors [45].

### Protein immunoprecipitation

For small RNA capture assays, 1x10^7^ cells stably expressing V5-eGFP, V5-Ago2 or V5-Piwi4 were infected with ZIKV PE243 at MOI 1. At 48 hpi the cells were scraped and washed with PBS. A detailed protocol has been described previously [41]. In short, cells were resuspended in lysis buffer (150mM NaCl, 5mM MgCl2, 20mM Hepes (pH 7.4), 0.5% Triton X-100, protease inhibitor cocktail [Roche]) and kept on ice for 20 min, followed by centrifugation at 15000 x g at 4°C for 25 min. The supernatant was then transferred into fresh tubes on ice and mouse anti-V5 antibody (at 1:500; ab27671 Abcam) added. Tubes were rotated for 2 h at 4°C. Following this, protein G magnetic beads (Dynabeads Protein G, Life Technologies) equilibrated with cold washing buffer (150mM NaCl, 5mM MgCl_2_, 20mM Hepes (pH 7.4), 0.5% Triton X-100) were added. Tubes were again rotated for 1 h at 4°C. The beads were then washed 4 times with washing buffer. Washed beads were finally resuspended in 100 μl washing buffer and 1/20 of the volume was subjected to western blot analysis while the remainder was used for RNA extraction.

### Extraction of protein-bound small RNA from beads

5 μl of proteinase K (20mg/ml) was added to magnetic beads resuspended in washing buffer and samples were placed into a water bath at 37°C for 30 min. Following this, 1 ml of Trizol reagent (Life Technologies) was added to the sample and processed as per the manufacturer’s instructions.

### cDNA synthesis

250 μl Trizol was added to 1.8x10^5^ AF5 or AF319 cells per well of a 24-well plate. Material from two wells was then pooled and the total cellular RNA extracted as per the manufacturer’s instructions. 1.5 μg of total RNA was used for cDNA synthesis using Superscript III (Life Technologies) and oligo(dT)15 (Promega), for quantification of mRNAs, or random primers (Promega), for quantification of ZIKV genomic RNA, according to manufacturer’s instructions.

### Small RNA sequencing and sequence analysis

**Fig 1:**
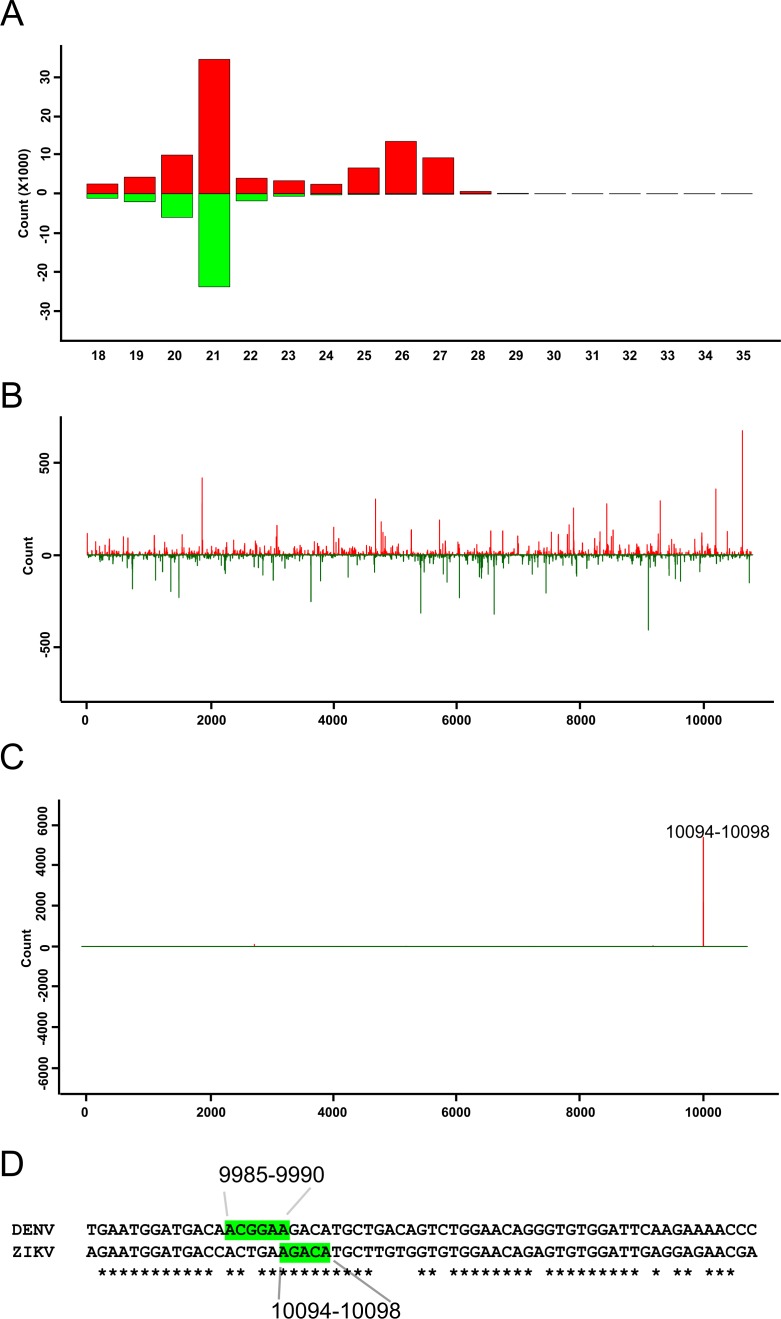
ZIKV-specific small RNAs in *Ae*. *aegypti-*derived Aag2 cells. Size distribution of small RNA from ZIKV (MOI 1, 48 hpi) infected cells. **(A)** Sequences mapping to viral genome (red) and antigenome (green). Distribution of 21 nt **(B)** or 27 nt **(C)** long small RNAs along the ZIKV genome (red, positive numbers on Y-axis) or antigenome (green, negative numbers on Y-axis), the number next to peak indicates the location of piRNAs. Results shown are for one representative experiment, out of two performed. **(D)** Partial alignment of DENV and ZIKV genomes is shown, green boxes indicate positions 9985–9990 (starting locations for most predominant DENV-derived piRNAs) or positions 10094–10098 (starting locations of ZIKV-specific piRNAs).

Trizol was added to 1x10^6^ cells or immunoprecipitation samples and RNA was extracted according to the manufacturer’s protocol. To increase small RNA precipitation efficiency, glycogen was added as a carrier. Small RNAs of 15–40 nt in length were gel purified and sequenced on an Illumina HiSeq 4000 at BGI Tech. Sequence reads were aligned to the ZIKV reference genome (Genbank accession number: KX197192.1) using in-house BLAST guided bioinformatics pipeline. Maximum of one mismatch was allowed in the alignments. Reads that matched the reference genome with alignment lengths from 18bp to 36bp were extracted for further analysis. They were separated into two groups, positive and negative based on the reads’ alignment to genome and antigenome respectively. Size distribution of aligned small RNAs is shown in Fig 1. See S1 Table for additional information. Small RNA sequencing data available at Sequence Read Archive (https://www.ncbi.nlm.nih.gov/sra) under accession PRJNA396680.

### qRT-PCR

Quantitative RT-PCR for ZIKV and the housekeeping gene S7 was performed using specific primers (S2 Table), SYBR green Mastermix (Abi) and an ABI7500 Fast according to manufacturer’s protocol. Results were analyzed using the ΔΔCt method.

### Transfection of nucleic acids

For transfections, 2 μl of Dharmafect 2 (GE Healthcare) per well of a 24 well plate was used. For RNAi reporter assays in SFV-infected cells, 1 ng of dsRNA (Fluc specific or LacZ specific) or 1 ng siRNA (targeting Fluc or Hygromycin B resistance gene) were co-transfected with 100 ng of pIZ-Fluc. For assessing silencing efficiency in ZIKV-infected cells, 200 ng of pIZ-Fluc and 100 ng of pAc1-Rluc plasmids were co-transfected with 0.25 ng of dsRNA (Fluc specific or eGFP specific) or 0.25 ng of siRNA (targeting Fluc or Hygromycin B resistance gene). To induce mosquito host gene silencing, 300 ng of gene-specific dsRNA was used.

### dsRNA production

T7 promoter-flanked PCR products were used for *in vitro* transcription. These were treated with DNase1 and RNaseA followed by column purification of the dsRNA using the RNAi Megascript kit (Life Technologies) as per the manufacturer’s instructions. For silencing purposes, previously described and verified dsRNAs were used [24,31,36].

### Luciferase assays

Aag2, AF5 and AF319 cells were seeded at 1.5x10^5^ cells per well in 24 well plates and infected with either SFV(3H)-*FFLuc*, SFV6(3H)-*RLuc*-2SG-p19, SFV6(3H)-*RLuc*-2SG-ZIKA_C, SFV6(3H)-RLuc-2SG-eGFP. Cells were lysed with passive lysis buffer (Promega). Luciferase Assay System (Promega) was used to measure firefly luciferase activity only. To measure *Renilla* activity, the *Renilla* luciferase Assay (Promega) system was used. For measuring both activities the Dual Luciferase Reporter Assay System (Promega) was utilized.

### Protein sequence comparison

To determine the similarity between the capsid protein of different flaviviruses, Needleman-Wunsch algorithm was used (http://www.ebi.ac.uk/Tools/psa/emboss_needle/). The Genbank access numbers are as follows: KX197192.1 (ZIKV), KM204118.1 (dengue virus [DENV]), KF769016.1 (YFV).

## Results

### ZIKV infection induces the production of virus-derived small RNAs in *Ae*. *aegypti*-derived mosquito cells

To assess the production of virus-derived small RNAs Aag2 cells were infected with a Brazilian ZIKV isolate, PE243 [45] at a multiplicity of infection (MOI) of 1. RNA was isolated at 48 hours post-infection (hpi) and small RNA pools were sequenced and analyzed. ZIKV-specific 21 nt long siRNAs (vsiRNAs) were found to be produced in infected cells (Fig 1A). Furthermore, the number of vsiRNAs mapping to the ZIKV genome were found to be roughly equal to those mapping to the antigenome with the vsiRNAs being distributed across the genome and antigenome (Fig 1B). Small RNAs in the size range of ZIKV-specific vpiRNAs (25–29 nts) were also detected. Positional mapping indicated that one region near the 3’ end of the genome gave rise to the majority of vpiRNAs as their 5’ ends mapped to positions 10094–10098 (Fig 1C), a region in the NS5-encoding region. Furthermore, these vpiRNAs were exclusively of positive polarity. Additional sequencing analysis of these putative vpiRNAs molecules showed a lack of the typical piRNA ping-pong signature, i.e. the absence of U1/ A10 predominance in the small RNA sequences. Intriguingly, DENV-specific vpiRNAs also mapped to a small number of locations on the genome [35]. Comparative analysis showed that the most predominant piRNA sequences of DENV (piRNA 5’ ends mapping to position 9985–9990) overlap with this ZIKV-specific, piRNA-like small RNA generating region (Fig 1D).

### Analysis of the interactions between virus-derived RNAs and RNAi effector proteins

**Fig 2:**
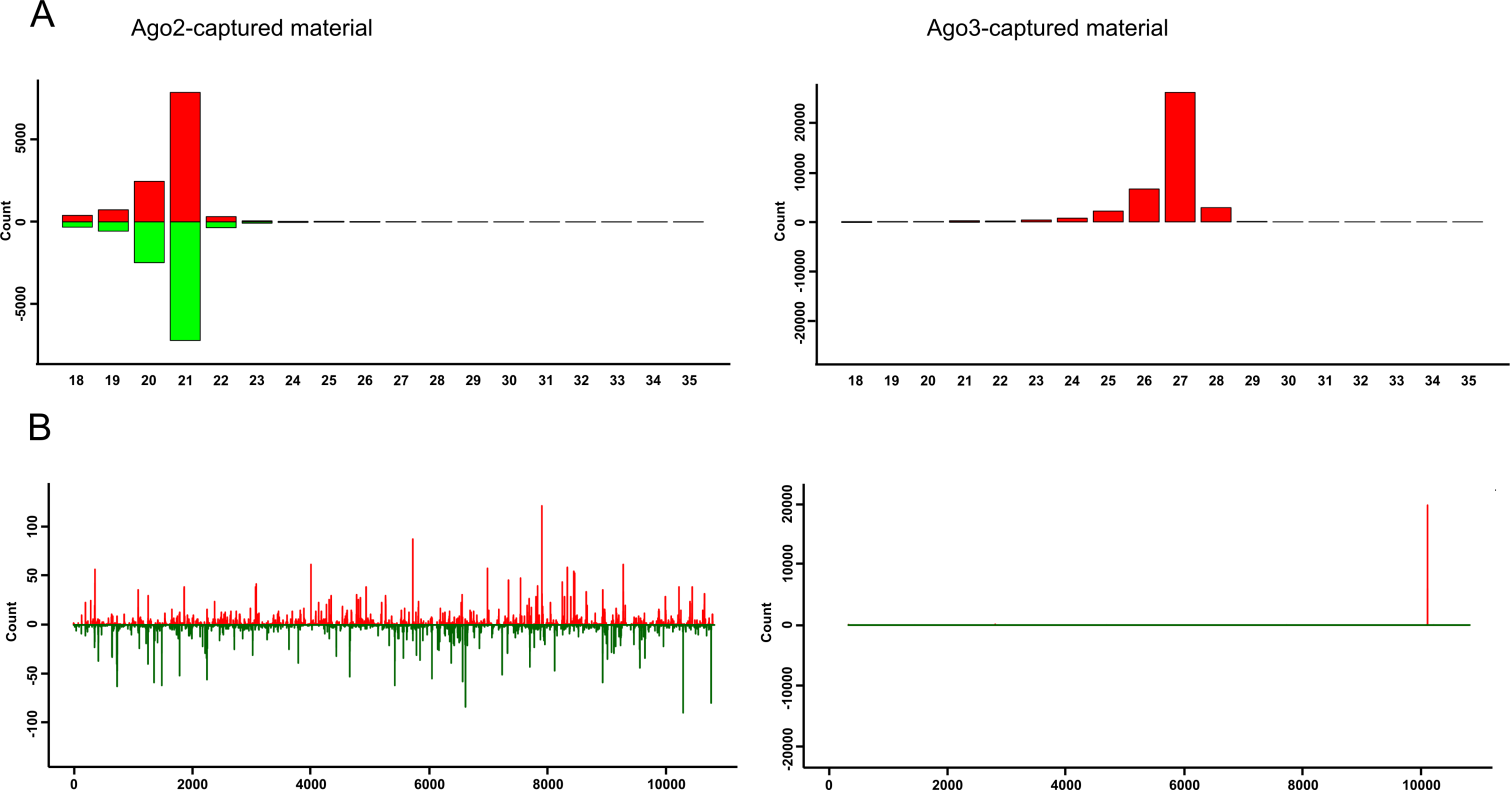
Characterization of ZIKV-specific small RNAs bound by Ago2 or Ago3. Aag2 cells expressing V5-Ago2 or V5-Ago3 were infected with ZIKV (MOI 1). At 48 hpi V5-tagged protein was immunoprecipitated followed by the isolation of small RNAs bound to these proteins. Analysis of Ago2 (left panels) and Ago3 (right panels) associated small RNAs are shown. **(A)** Size distribution of small RNAs mapping to ZIKV genome (red) or antigenome (green). **(B)** Distribution of Ago2 and Ago3 bound small RNAs (21 nt and 27 nt, respectively) along the ZIKV genome (red, positive numbers on Y-axis) or antigenome (green, negative numbers on Y-axis). Two independent experiments were carried out and the results of one representative experiment are shown here.

We analyzed whether virus-derived small RNAs are loaded into RNAi effector proteins and determined which of these would be crucial for mediating antiviral activities. Ago2-bound small RNAs were investigated using a previously developed Aag2 cell line which stably expresses V5-tagged Ago2 [41]. Ago2 was immunoprecipitated (S1 Fig) at 48 hpi following infection with ZIKV (MOI 1) and the captured small RNAs were sequenced. Samples from cells expressing V5-tagged eGFP were used as control. Small RNA analysis showed that Ago2 bound 21 nt small RNAs (Fig 2) and these ZIKV-specific vsiRNAs were distributed along both genomic and antigenome strands. This indicates that dsRNA-derived vsiRNAs were successfully loaded into Ago2. Similar experiments were conducted to determine which RNAs were bound by Ago3, Piwi4, Piwi5 or Piwi6 proteins (Fig 2, S2 Fig), which are involved in the piRNA ping-pong amplification loop in *Ae*. *aegypti* [34,46]. Sequence analysis revealed Ago3-bound ZIKV genome vpiRNAs mapped specifically to positions 10094–10098 of the viral genome (Fig 2B), while the read numbers for other potential vpiRNAs were too low for further analysis. However, no ZIKV-specific piRNA molecules were found to be associated with either Piwi5 or Piwi6 (S2 Fig), although small amounts of 18 nt and 19 nt small ZIKV-specific RNAs were found to be associated with these proteins. In the case of Piwi4, relatively low amounts of ZIKV-specific vsiRNA and vpiRNA-like small RNAs were detected (S2 Fig). However, these may not be bound by Piwi4 directly as other RNAi pathway proteins (Ago2, Dcr2, Ago3, Piwi5 and Piwi6) can interact with Piwi4, as previously shown [34,41], and this will require further investigations. Several flaviviruses are known to produce miRNA-like small RNAs (22–24 nt) and thereby regulate host gene expression [47]. However, our data indicated that the majority of small RNAs in the size range of 22–24 nt could be mapped to positions 10094–10098 (S3 Fig) and these molecules were bound by Ago3, suggesting that these small RNAs were related to vpiRNAs rather than miRNAs.

### The effect of RNAi effector knockdown on ZIKV replication

**Fig 3:**
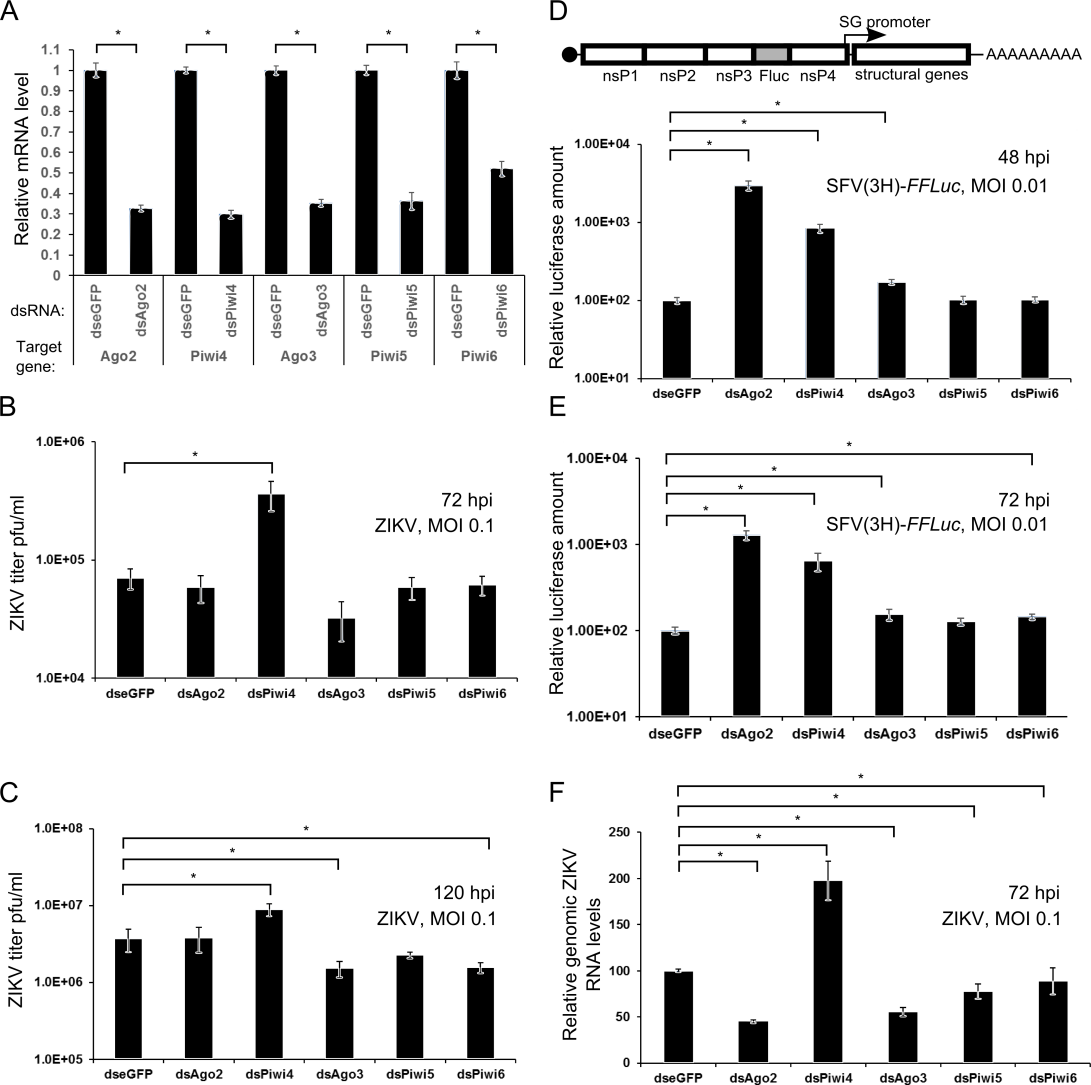
Effect of RNAi effector knockdown on ZIKV replication. **(A)** Aag2 cells were transfected with dsRNAs targeting Ago2 (dsAgo2), Piwi4 (dsPiwi4), Ago3 (dsAgo3), Piwi5 (dsPiwi5), Piwi6 (dsPiwi6) or eGFP (dseGFP, control). After 24 h the total cellular RNA was isolated and subjected to cDNA synthesis. Using gene-specific primers the knockdown efficiency was assessed by qRT-PCR. The mean values of relative RNA levels from three independent experiments together with standard error are shown. **(B, C)** Aag2 cells transfected with dsRNAs were infected with ZIKV (MOI 0.1), samples were collected for titration at 72 hpi **(B)** or 120 hpi **(C)**. When samples were collected at 120 hpi, the infected cells were re-transfected with dsRNAs at 48 hpi. The mean values from 6 independent experiments together with standard error are shown. **(D, E)** Transfected cells were infected firefly luciferase-expressing SFV(3H)-*FFLuc* virus (depicted above) at MOI 0.01 and cells were lysed at 48 hpi **(D)** or 72 hpi **(E)** to measure luciferase activities. The relative mean firefly luciferase amounts with error of the mean, from two independent experiments conducted in quadruplicate are shown. **(F)** Mean relative ZIKV genomic RNA levels from three independent experiments (using ribosomal S7 as a housekeeping gene) in dsRNA treated cells at 72 hpi, transfection conducted as described in panel B. Error bars show error of mean. For statistical analysis, to reduce the heterogeneity, log-transformation (B, C, E) or 1/x-transformation (D, F) was conducted; Dunnett’s test was used for multiple comparisons purposes; * indicates significance by p<0.05.

We previously showed that the knockdown of Ago2 and Piwi4 enhanced replication of the alphavirus SFV and the orthobunyavirus BUNV in Aag2 cells [24,31]. Similarly, others have shown that silencing of Ago2 in *Ae*. *aegypti*-derived cells or *Ae*. *aegypti* mosquitoes enhances Sindbis virus (SINV; genus *Alphavirus*) and dengue virus (DENV, a flavivirus related to ZIKV) replication [23,35,48]. However, in the case of DENV this was not found to be statistically significant. In order to assess the antiviral roles of these known RNAi effectors on ZIKV, cells were first transfected with previously validated dsRNAs (see Materials and Methods) targeting RNAi effector proteins (Fig 3A) followed by infection with ZIKV at MOI 0.1 24 h later. For validation of the assay, Firefly luciferase (*FFLuc*) expressing SFV vector SFV(3H)-*FFLuc* was used. Surprisingly, we found that the knockdown of Ago2 had no effect on ZIKV titer at 72 hpi or 120 hpi (Fig 3B and 3C). However, the knockdown of Piwi4 significantly increased virus titers as measured at 72 hpi and 120 hpi time points. Additionally, silencing of Ago3 also resulted in decreased ZIKV titers at 120 hpi while silencing of Piwi5 or Piwi6 had no major effect on ZIKV production. However, in the case of SFV(3H)-*FFLuc* luciferase readings at 48 hpi indicated that Ago2 and Piwi4 were antiviral, as luciferase expression was 25 and 8 fold higher respectively (Fig 3D). Similarly, at 72 hpi, where the reads were 12 and 6 fold higher for Ago2 and Piwi4 respectively (Fig 3E). Silencing of Ago3, Piwi5 or Piwi6 had no major effect on luciferase levels, as expected [24] (Fig 3D and 3E).

**Fig 4:**
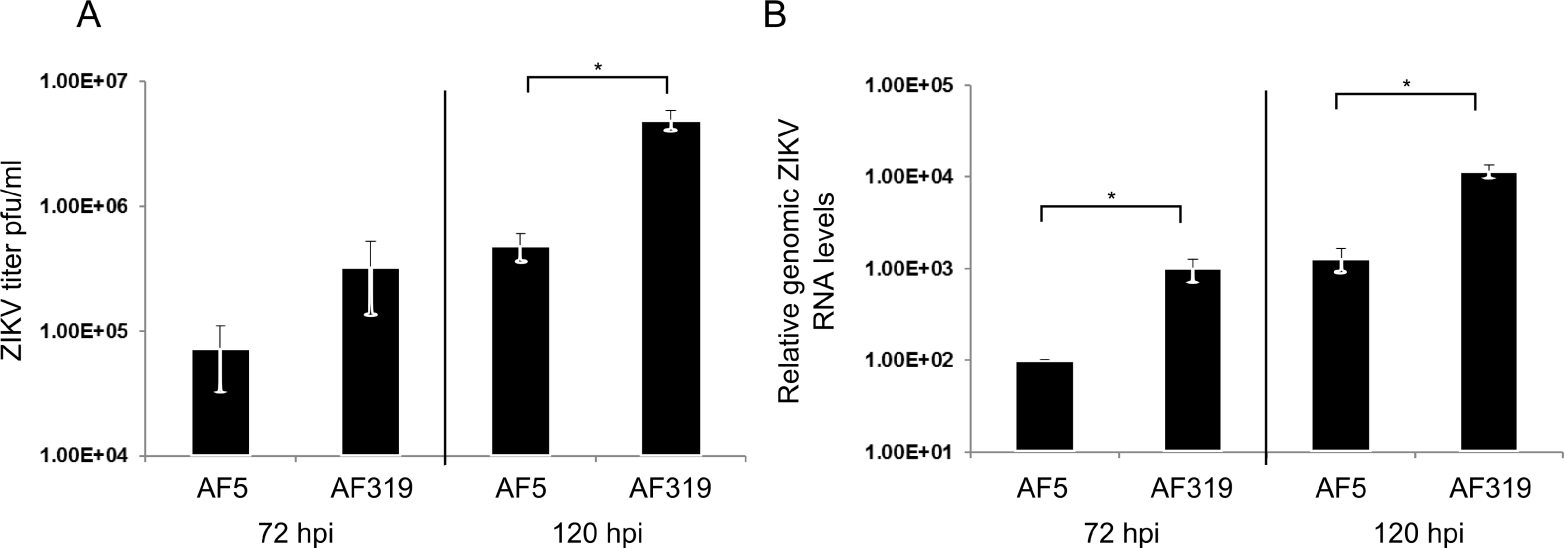
Replication of ZIKV in Dcr2 knockout mosquito cells. AF319 (Dcr2 deficient) cells or their parental cells AF5 (derived from Aag2 cells) were infected with ZIKV (MOI 0.1) and virus in supernatant titrated at 72 hpi and 120 hpi **(A)**. The mean values of 6 independent experiments together with error of mean are given. Alternatively, relative viral genome copy numbers in the cells were measured by qRT-PCR using the S7 gene as a housekeeping gene. Obtained values were normalised to those in AF5 cells at 72 hpi **(B)**. The mean values of three independent experiment together with error of mean are shown. * indicates significance by two-tailed Student t-test assuming unequal variance, p<0.05.

The effect of the knockdown of RNAi effector proteins on ZIKV replication was also assessed by quantifying the levels of viral genomic RNA present in infected cells. This was achieved by qRT-PCR which indicated that, surprisingly, silencing of Ago2 resulted in lower levels of viral genomic RNA (Fig 3F). Knockdown of Piwi4 was found to enhance virus replication while silencing of Ago3 had an inhibitory effect, suggesting that Ago3 has proviral activity. These data correlated with virus titers (Fig 3). The unexpected failure of Ago2 silencing to permit increased ZIKV replication led us to verify the role of the exo-siRNA pathway through the use of Dcr2 knockout cells. AF5 cells are derived from a single clone of Aag2 cells which possess functional Dcr2 activity and subsequent siRNA production. Conversely, CRISPR-Cas9 technology was used to produce the Dcr2-deficient AF5 derivative cell line, AF319, which does not generate siRNAs, as described previously [41]. A comparison between the replication of ZIKV in both AF5 and AF319 cell lines showed higher virus production in AF319 at 72 and 120 hpi (Fig 4A). Moreover, the results of the qRT-PCR experiment to measure viral genomic RNA levels showed that the lack of Dcr2 benefits virus replication (Fig 4B). This indicated that Dcr2 can recognize and cleave ZIKV-derived dsRNA and the exo-siRNA pathway does play an antiviral role against ZIKV.

**Fig 5:**
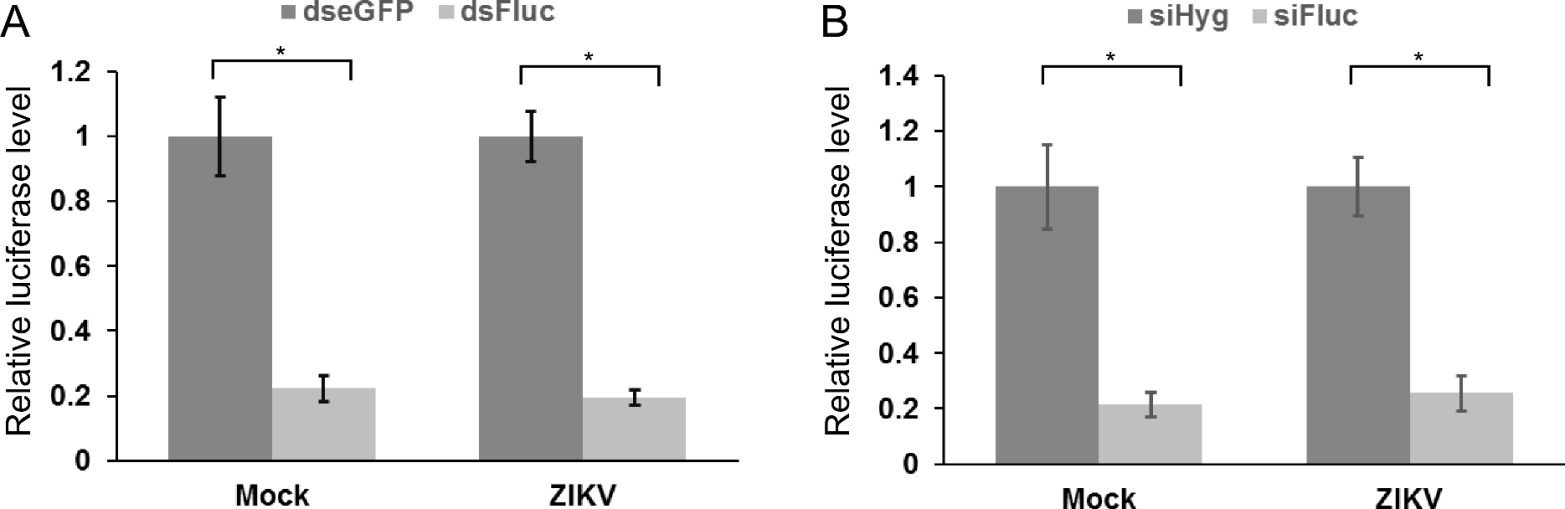
Effect of ZIKV infection on gene silencing. Aag2 cells were either mock-infected or infected with ZIKV at MOI 1. **(A)** At 96 hpi firefly luciferase (Fluc) and Renilla luciferase (Rluc) expressing vectors were co-transfected with dsRNA targeting Fluc (dsFluc) or as control, eGFP (dseGFP); relative luciferase levels are shown on the Y- axis (with Fluc/Rluc ratio in dsLacZ transfected cells set to 1). **(B)** Alternatively, siRNAs against Fluc (siFluc) or Hygromycin B resistance gene (siHyg) as a control were used in co-transfection; cells were lysed at 24 h post-transfection. Mean values with standard error are shown for three independent experiment conducted in triplicate. * indicates significance p<0.05, according to two-way ANOVA.

Since insect and plant viruses can inhibit RNAi pathways [49], we assessed if ZIKV can interfere with siRNA-based silencing. For this, pIZ-Fluc and pAc1-Rluc plasmids were co-transfected together with dsRNAs targeting firefly luciferase or eGFP (control) into mock- or ZIKV- infected (MOI 1) Aag2 cells at 96 hpi. In mock-infected and ZIKV-infected cells the silencing was 5-fold, which indicates that ZIKV infection did not reduce silencing efficiency (Fig 5A). Similarly, by using siRNAs targeting firefly luciferase or Hygromycin B resistance gene (negative control), we observed the same outcome as siRNAs targeting Fluc functioned as efficiently in mock and ZIKV-infected cells (Fig 5B). These data indicated that ZIKV did not inhibit the siRNA pathway.

### Effect of ZIKV capsid (C) protein on antiviral RNAi

A recent report has determined that the expression of the C protein of the related mosquito-borne flavivirus, YFV, can enhance the replication of SINV. It was shown to bind viral dsRNA and protect nucleic acid from cleavage by human dicer [40]. The replication-enhancing effect was found to be common for the C protein of other flaviviruses, including ZIKV. However, no further studies were conducted with these. The sequence similarity between ZIKV and YFV capsid is only 47.2% (identity 24.8%), as determined by the Needleman-Wunsch algorithm.

**Fig 6:**
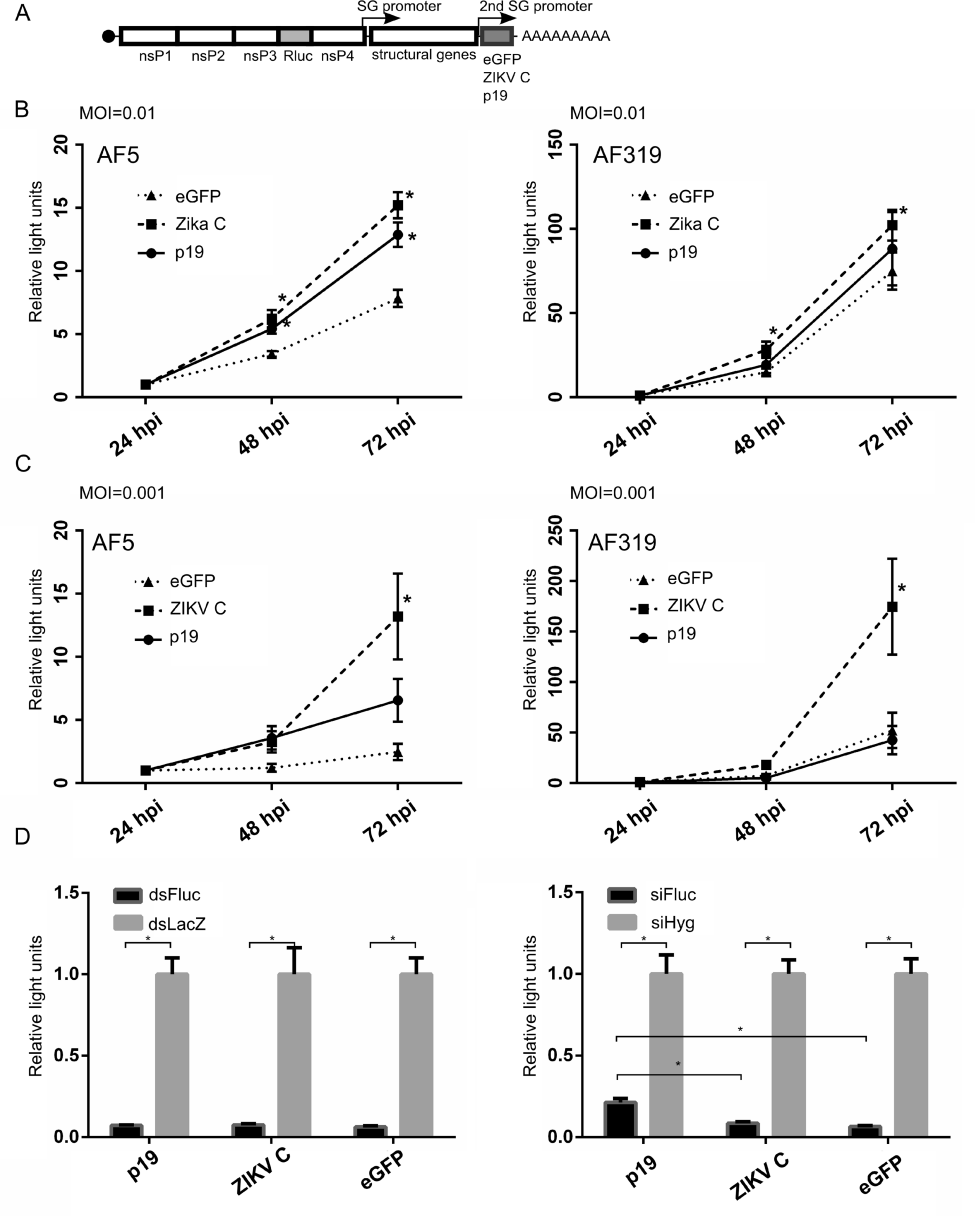
ZIKV capsid C enhances replication of a reporter SFV but does not affect the exo-siRNA pathway. **(A)** Design of Rluc reporter SFV expressing eGFP, p19 or ZIKV C protein from a duplicated subgenomic (2SG) promoter: SFV6(3H)-*RLuc*-2SG-eGFP, SFV6(3H)-*RLuc*-2SG-p19 or SFV6(3H)-*RLuc*-2SG-ZIKV_C respectively. These viruses were used to infect AF5 or AF319 cells at either MOI 0.01 **(B)** or MOI 0.001 **(C)**. Experiments were performed three (B) or four (C) times in quadruplicate. Replication and/or spread was determined by *Renilla* luciferase activity. The mean relative luciferase activity values (compared to the levels measured at 24 hpi) are given on the Y-axis and the error bars indicate the error of the mean. **(D)** At 24 hpi, Fluc-expressing reporter plasmid was co-transfected with dsRNAs (against Fluc, dsFluc; or LacZ, dsLacZ) or siRNAs (against Fluc, siFluc; or Hygromycin B resistance gene, siHyg) into cells infected with viruses described under (A) at MOI 1. Cells were lysed at 24 h post-transfection. The relative mean Fluc activity values, together with error of the mean, from three experiments conducted in quadruplicate are shown. * indicates significance, p<0.05. For panels B and C, Student t-test (two-tailed, assuming unequal variance); for D, two-way ANOVA.

Despite that we could not detect that ZIKV infection affects dsRNA or siRNA-mediated silencing in a plasmid-based assay (see above, Fig 5), we decided to assess the hypothetical possibility that ZIKV C protein could also, similar to YFV, inhibit the antiviral siRNA response. We started by cloning the full length ZIKV C (amino acid residues 1–122) under the duplicated subgenomic promoter of SFV. Recombinant SFV expressing either tombusvirus siRNA-binding p19 [50] or eGFP were used as positive and negative controls, respectively. In addition, these viruses expressed *Renilla* luciferase reporter inserted into duplicated nsP2 cleavage sites situated between non-structural nsP3 and nsP4 (Fig 6A). Infection of AF5 or (Dcr2 KO) AF319 cell lines at low MOI (0.01) was carried out to monitor virus replication and/or spread by determining *Renilla* luciferase activity. Although the replication of SFV expressing ZIKV C was enhanced in AF5 cells as expected (Fig 6A), surprisingly, enhanced replication was also observed in Dcr2 deficient AF319 cells. This contrasts to results with p19 where its enhancing effect in AF319 cells compared to AF5 cells is lost (Fig 6B). The effect of the expression of ZIKV C was found to be even more prominent and evident at lower a MOI (0.001) (Fig 6C). Both ZIKV C and p19 benefitted SFV replication in AF5 cells, with the former promoting greater virus replication compared to p19. Similarly, in AF319, the effect of Zika C on SFV replication was strong and Rluc levels were again significantly higher compared to viruses expressing p19 or eGFP. These results indicated that the ZIKV C protein could help to overcome inhibitory antiviral processes and enhance SFV replication and/or spread more efficiently than p19, even in the absence of a functioning exo-siRNA pathway.

To determine whether the ZIKV C protein could inhibit the exo-siRNA pathway, Aag2 cells were infected (MOI 1) with recombinant SFV expressing ZIKV C, p19 or eGFP as described above. At 24 hpi cells were transfected with a firefly luciferase (Fluc)-expressing plasmid along with Fluc-specific (or negative control) dsRNAs/siRNAs. When dsRNAs were transfected, determination of Fluc activity at 24 h post-transfection showed that neither the expression of ZIKV C nor p19 affected the silencing efficiency, as compared to eGFP (Fig 6D). Conversely, p19 expression led to reduced siRNA-mediated silencing activity, as expected, as approximately 20% luciferase activity in p19 was observed versus 5% activity in eGFP expressing cells. However, the expression of the ZIKV C protein had only a minor effect with 7% activity of Fluc remaining. Thus, this suggested that the ZIKV C protein has no effect on small RNA pathway-mediated gene silencing, which correlates well with the notion that in ZIKV infected cells no general siRNA pathway inhibition occurs (Fig 5).

## Discussion

The production of virus-specific siRNAs and piRNA-like molecules in infected mosquitoes or mosquito-derived cells has been demonstrated for viruses from all of the major arbovirus families: *Flaviviridae*, *Togaviridae* and *Bunyaviridae* [6,7]. Here we demonstrated that ZIKV also induces the production of 21 nt vsiRNAs as well as small RNAs of the size expected for piRNAs; although these do not display the molecular signature of typical piRNAs. We found that ZIKV-specific vsiRNAs were loaded into Ago2 and thus, in principle, can target viral RNA and mediate degradation. Surprisingly however, the knockdown of Ago2 was not sufficient to increase ZIKV replication and only the knockout of Dcr2 led to an increase in virus replication. This suggests that the exo-siRNA pathway does mediate antiviral activity against this virus; however, its replication may be protected from Ago2 mediated activity through a yet unknown virus-mediated resistance mechanism. This may not be so exceptional as it has previously been observed that the knockdown of Ago2 only resulted in an approximately 2-fold (statistically non-significant) increase in DENV genomic RNA in Aag2 cells [35]. Similarly, Ago2 silencing was only found to be marginally beneficial (20%) for BUNV replication [31], but another orthobunyavirus -Schmallenberg virus- showed a 7-fold increase in viral RNA levels. Perhaps direct dicing of viral dsRNA is more relevant in this context than RISC-mediated antiviral effects. It is also known that flavivirus replication is coupled to viral packaging [51]. Our data could suggest that the packaging of ZIKV RNA occurs rapidly which prevents the recognition of genomic RNA by Ago2 loaded with specific siRNAs. ZIKV is known to heavily modify the ER and form replication factories in mammalian cells [52]. Importantly in the context of RNAi, West Nile Virus (WNV) was shown to become resistant to siRNAs after infection of vertebrate cells following transfection but not electroporation, suggesting that evasion of silencing was taking place [53]. Thus, it could be expected that similar membrane modifications occur in insect cells and such replication factories could shield ZIKV genome from Dcr2 and/or Ago2 detection. This is also relevant from a comparative point of view, as alphavirus replication (despite also occurring in cytoplasmic, membranous structures) is generally increased by Ago2 silencing. How RNAi effectors target viral replication complexes is therefore an intriguing question and localization studies are required to shed light on these processes and differences between virus families. Thus, there are common points and differences between arbovirus families with regards to RNAi responses in mosquito vectors, and the functional relevance of these need to be further assessed in comparative studies.

To our knowledge, no study has shown that vpiRNAs directly inhibit virus replication and the role of PIWI proteins remains enigmatic. Indeed, only the Piwi4 protein has consistently shown antiviral activity against bunyaviruses and alphaviruses [24,31]. However, the antiviral effector mechanism of Piwi4 is unknown. It may interact with piRNA-like small RNAs or 21 nt small RNAs directly, or this association may occur via Piwi4 interaction partners Ago3, Piwi5, Piwi6 and Ago2. Moreover, Piwi4 is not required for the production of virus-specific piRNAs, at least in the case of alphaviruses and DENV [24,35]. Although we could detect the production of ZIKV-specific piRNA-sized small RNAs, these could be mapped to a single site on the viral genomic strand and lacked the characteristic piRNA signature. Those piRNA-like molecules or vpiRNAs were found to be bound by Ago3; although, intriguingly the knockdown of Ago3 decreased ZIKV replication slightly. Similarly in the case of DENV, vpiRNAs mapped to a few discrete sites on the genome and Ago3 was also found to be proviral [35]. In another study, Piwi5, a key protein involved in piRNA production, was found to positively affect BUNV virus [31], which further questions the antiviral role of piRNAs, at least during the acute phase of infection. Intriguingly, a recent study [54] assessed the induction of virus-derived small RNAs following infection of *Ae*. *aegypti* by ZIKV up to 14 days post-infection and, similarly, virus-specific piRNA-like molecules did not display the ping-pong signature. However, over time piRNAs could be mapped on more locations on the ZIKV genome. The relevance of these observations needs to be experimentally assessed, especially as a study in Aag2 cells infected with mosquito-borne RVFV also showed an increase in vpiRNAs (with the ping-pong signature) especially from the S and M genome segments over time [21]. The role and activity of the piRNA pathway may be delayed compared to the exo-siRNA pathway.

Since flaviviruses encode miRNAs to control host cell gene expression [47], it is possible that ZIKV, or flaviviruses in general, use piRNAs for this purpose, although this needs further experimental verification. Regardless, the direct role of vpiRNAs remains to be elucidated and the function of Piwi4 in this or other pathways remains perplexing, despite its consistent antiviral activity.

The ability of the flavivirus C protein to inhibit small RNA-based antiviral responses is intriguing. The YFV C protein was found to bind both single-stranded RNA and dsRNA, and prevented cleavage of the latter [40] in an *in vitro* experiment with human Dicer. Furthermore, expression of the C protein from other flaviviruses (WNV, Rio Bravo virus, ZIKV, DENV) enhanced SINV replication. However, the lengths (or specific amino acid residues) of the expressed flavivirus C proteins were not indicated in the published study and so it was not possible to determine if longer, membrane-bound or shorter, cleaved C proteins were mediating the observed effects [55]. As shown here, the full length ZIKV C protein could also enhance the replication of another alphavirus, SFV. However, this also occurred in cells lacking Dcr2, which suggests that the ZIKV C protein inhibits antiviral processes other than the exo-siRNA pathway (Fig 5). YFV is phylogenetically more distant than DENV to ZIKV, and although the general topology of the C protein is conserved among the three viruses [55], the sequence similarity between YFV and ZIKV C proteins is low (24.8%). Thus, it is likely that their proviral mode of action is different. Moreover, in ZIKV-infected cells no reduction in the efficiency of siRNAs or dsRNAs to silence gene expression could be detected, which also suggests that the ZIKV C protein acts in a manner that does not involve the counteraction of Dcr2.

Understanding of virus-vector relations provides further insights into the spread of disease. Recent studies by us and others indicate a complex interplay between arboviruses and mosquito host responses. Viruses have adopted countermeasures against the immune system, which in turn results in selective pressures on the vector. It is highly possible that relative resistance to the antiviral effect of Ago2 is a result of this process. Future work on flaviviruses, such as ZIKV, will need to identify whether any direct targeting of this effector takes place, but also why vpiRNA characteristics are different to other arbovirus families. This is likely to give important clues relating to how different arboviruses are spread by mosquitoes and whether family-specific weaknesses can be exploited.

## Supporting information

S1 Table Information regarding small RNA sequencing data. Number of ZIKV-specific sequencing reads obtained by analyzing small RNAs in total cellular RNA samples or in samples that were captured by pulldown of V5-tagged eGFP, Ago2, Ago3, Piwi5, Piwi6 and Piwi4 from Aag2 cells.
(DOCX)

S2 Table PCR primers used in the study. (DOCX)

S1 Fig Immunoprecipitation of siRNA and piRNA pathway proteins. Immunoblot analysis of the immunoprecipitation (IP) samples obtained from Aag2 cell lines infected with ZIKV (MOI 1) 48 h p.i. IP of V5 tagged eGFP, Ago2, Ago3, Piwi5, Piwi6 and Piwi4 was conducted using magnetic beads carrying anti-V5 antibody.
(TIF)

S2 Fig Characterization of ZIKV-specific small RNAs captured by Piwi5, Piwi6 or Piwi4. V5-tagged Piwi5 or Piwi6 expressing cells were infected with ZIKV (MOI 1). At 48 hpi they were subjected to immunoprecipitation via V5-tag specific antibody. Analysis of Piwi5 **(A)** or Piwi6 **(B)**, Piwi4 **(C)** associated small RNAs indicated the size distribution of those mapping to the ZIKV genome (red) or antigenome (green). Two independent experiments were carried out and the results of one representative experiment are shown here.
(TIF)

S3 Fig Zika-specific small RNA with size 22-24nt. The distribution of 22, 23 or 24 nt long small RNA along the ZIKV genome (red, positive numbers on Y-axis) or antigenome (green, negative numbers on Y-axis). Analysis of total RNA samples isolated from infected Aag2 cells **(A)** or analysis of RNA bound to Ago3, captured by immunoprecipitation from infected cells expressing V5-tagged Ago3 **(B).** Samples were collected 48 hpi from ZIKV (MOI 1) infected cells and the experiment was repeated twice. The results of one representative experiment are shown here.
(TIF)
