# Supplementary material for: Characterization of the Zika virus induced small RNA response in Aedes aegypti cells
Source: PLoS Negl Trop Dis. 2017 Oct 17;11(10):e0006010. doi: 10.1371/journal.pntd.0006010 (PMC5667879; doi:10.1371/journal.pntd.0006010)
Supplement: S1 Table — Number of ZIKV-specific sequencing reads obtained by analyzing small RNAs in total cellular RNA samples or in samples that were captured by pulldown of V5-tagged eGFP, Ago2, Ago3, Piwi5, Piwi6 and Piwi4 from Aag2 cells. (DOCX) [file pntd.0006010.s001.docx]

**S1 Table. Information regarding small RNA sequencing data.**

Number of ZIKV-specific sequencing reads obtained by analysing small RNA in total cellular RNA samples or in samples that were captured by pulldown of V5-tagged eGFP, Ago2, Ago3, Piwi5, Piwi6 from Aag2 cells.

| **Small RNA captured from Aag2 cells** | | | |
| --- | --- | --- | --- |
| **First experiment** | | | |
| **Pulled down protein** | **Number of ZIKV-specific 21 nt long reads** | **Number of ZIKV-specific 24-29 nt long reads** | **Number of total reads 18-35 nt** |
| V5-eGFP | 1 | 10 | 21809195 |
| V5-Ago2 | 14990 | 94 | 18855203 |
| V5-Ago3 | 300 | 38085 | 26295943 |
| V5-Piwi5 | 23 | 30 | 21366323 |
| V5-Piwi6 | 50 | 56 | 22377131 |
| V5-Piwi4 | 475 | 982 | 16596435 |
| **Second experiment** | | | |
| **Pulled down protein** | **Number of ZIKV-specific 21 nt long reads** | **Number of ZIKV-specific 24-29 nt long reads** | **Number of total reads 18-35 nt** |
| V5-eGFP | 8 | 24 | 4697405 |
| V5-Ago2 | 7338 | 61 | 19734674 |
| V5-Piwi5 | 10 | 8 | 25148045 |
| V5-Piwi6 | 55 | 9 | 21436734 |
| V5-Piwi4 | 363 | 219 | 28952913 |
| **Second experiment** | | | |
| V5-eGFP | 1 | 3 | 23659649 |
| V5-Ago3 | 193 | 3434 | 37122796 |
| **Analysis of total cellular RNA from ZIKV-infected Aag2 cells** | | | |
| **Cell line** | **Number of ZIKV-specific 21 nt long reads** | **Number of ZIKV-specific  24-29 nt long reads** | **Number of total reads 18-35 nt** |
| First experiment | 58299 | 30610 | 24893500 |
| Second experiment | 933 | 1432 | 33090425 |
