# Supplementary material for: Characterization of the Zika virus induced small RNA response in Aedes aegypti cells
Source: PLoS Negl Trop Dis. 2017 Oct 17;11(10):e0006010. doi: 10.1371/journal.pntd.0006010 (PMC5667879; doi:10.1371/journal.pntd.0006010)
Supplement: S2 Table — (DOCX) [file pntd.0006010.s002.docx]

**S2 Table. PCR primers used in the study.**

| **Primers for qRT-PCR** | |
| --- | --- |
| **Target** | **Forward/ Reverse primer** |
| ZIKV | GTTGTCGCTGCTGAAATGGA/GGGGACTCTGATTGGCTGTA |
| Ago2 | GGCTGCTCACCCAATGTATCAAGA/AACCGTTCGTTTTGGCGTTGAT |
| Piwi4 | CTTCTCCACCACAGCCAATG/GTCCAATCTGCCTGTTCTCCA |
| Ago3 | GCTTCGTTGAATGGTGACTACAC/TGACAGTTTGCCTTCTGGTAAG |
| Piwi5 | GAAGTTGAAATAACCACCAAAGAG/CCATCTACTACGCCGACTTTTC |
| Piwi6 | CGTTACTTACCAGAAATAGTGC/GGAAAATTCGTTCATCTTCCTT |
| S7 | CCAGGCTATCCTGGAGTTG/ GACGTGCTTGCCGGAGAAC |
