# Supplementary figures and images for: Characterization of the Zika virus induced small RNA response in Aedes aegypti cells
Source: PLoS Negl Trop Dis. 2017 Oct 17;11(10):e0006010. doi: 10.1371/journal.pntd.0006010 (PMC5667879; doi:10.1371/journal.pntd.0006010)

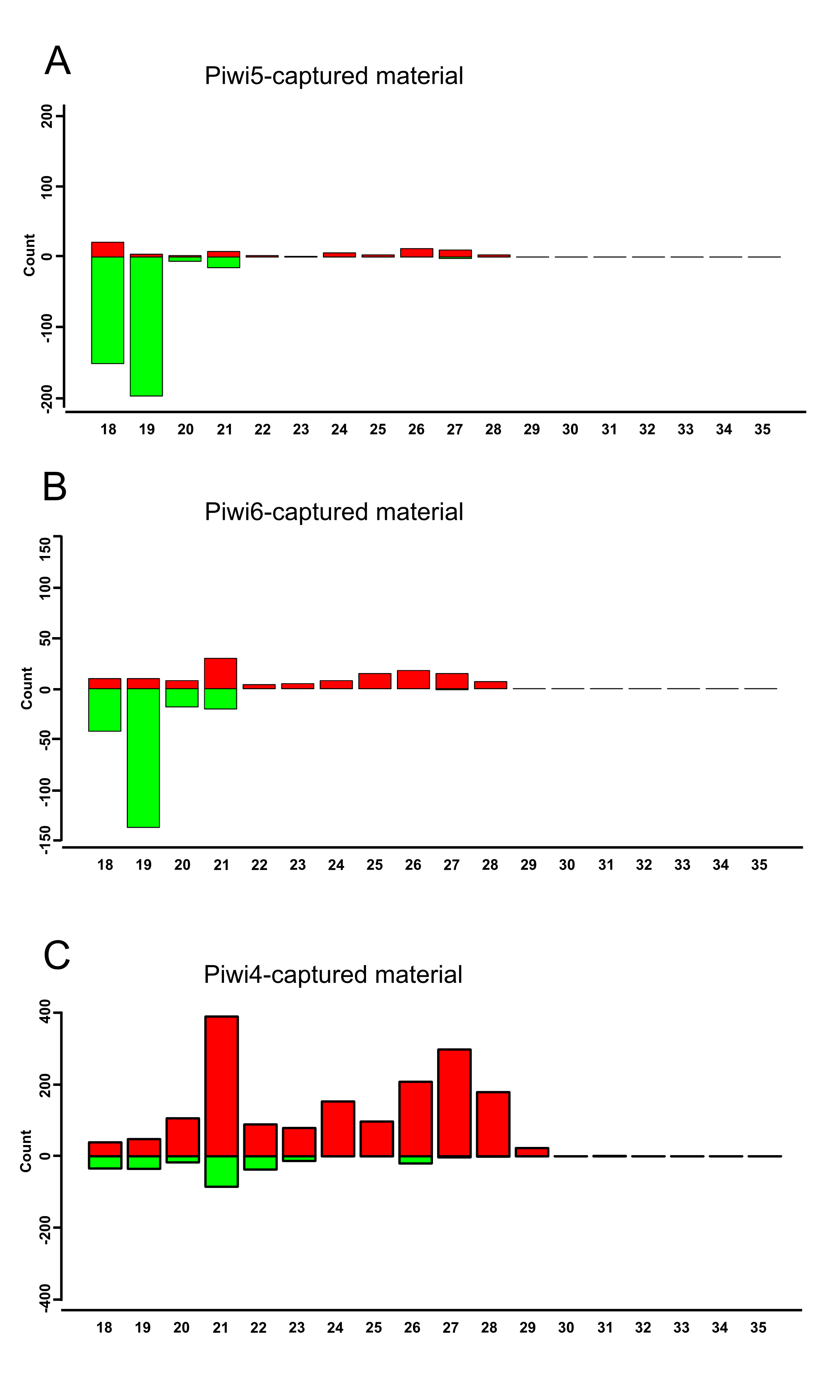

Supplement: S2 Fig — V5-tagged Piwi5 or Piwi6 expressing cells were infected with ZIKV (MOI 1). At 48 hpi they were subjected to immunoprecipitation via V5-tag specific antibody. Analysis of Piwi5 (A) or Piwi6 (B), Piwi4 (C) associated small RNAs indicated the size distribution of those mapping to the ZIKV genome (red) or antigenome (green). Two independent experiments were carried out and the results of one representative experiment are shown here. (TIF) [file pntd.0006010.s004.tif]

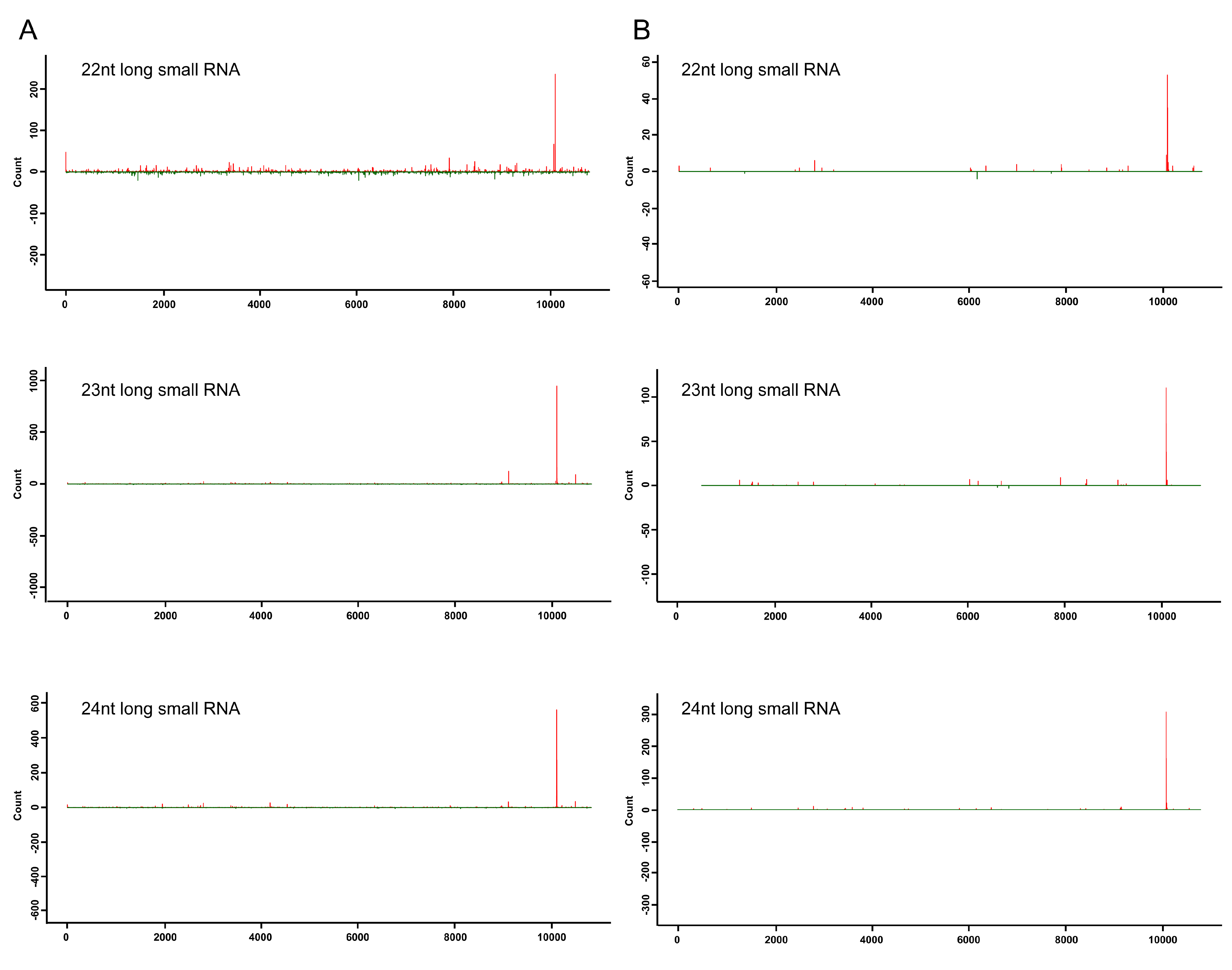

Supplement: S3 Fig — The distribution of 22, 23 or 24 nt long small RNA along the ZIKV genome (red, positive numbers on Y-axis) or antigenome (green, negative numbers on Y-axis). Analysis of total RNA samples isolated from infected Aag2 cells (A) or analysis of RNA bound to Ago3, captured by immunoprecipitation from infected cells expressing V5-tagged Ago3 (B). Samples were collected 48 hpi from ZIKV (MOI 1) infected cells and the experiment was repeated twice. The results of one representative experiment are shown here. (TIF) [file pntd.0006010.s005.tif]
